# Möbius edge band and Weyl-like semimetal flat-band in topological photonic waveguide array by synthetic gauge flux

**DOI:** 10.1515/nanoph-2023-0311

**Published:** 2023-07-14

**Authors:** Zhenzhen Liu, Guochao Wei, Huizhou Wu, Jun-Jun Xiao

**Affiliations:** College of Electronic and Information Engineering, Harbin Institute of Technology (Shenzhen), Shenzhen 518055, China; Shenzhen Engineering Laboratory of Aerospace Detection and Imaging, Harbin Institute of Technology (Shenzhen), Shenzhen 518055, China; School of Mathematical and Physical Sciences, Wuhan Textile University, Wuhan 430200, China

**Keywords:** artificial gauge field, beam dynamics, Möbius insulator, photonic waveguide, Weyl semimetal

## Abstract

The presence of *π* gauge flux enabled by positive and negative hopping amplitude can lead to Möbius bands, which was recently demonstrated in both realistic acoustic and photonic lattices, twisted at *k* = *π*. Here, we show that the artificial gauge flux configuration can be achieved by exploiting the interactions between photonic *s *and *p* orbital-like fundamental modes in circular and peanut-shaped waveguides, respectively. By manipulating the interplay between the gauge fields and the crystal symmetry, we show that breaking the primitive translation symmetry through lattice site dimerization and deformation can cause the original Dirac semimetal phase, characterized by a four-fold Dirac point at the Brillouin zone center, to transform into various topological phases. The designed photonic waveguide array supports topological phases such as Möbius insulator and Weyl-like semimetal phases. Noticeably different to the existing cases, we explicitly show that the twisting Möbius bands cross each other at *k* = 0 due to the lattice gauging with alternating sign, which results in distinct beam dynamics excitation. We also present Weyl-like flat-band edge states in such photonics waveguide arrays. Our results suggest that such *s* − *p *hybridized photonic waveguide array servers as a convenient and flexible platform for studying topological physics, particularly in simulating the effects of gauge field in alternative configuration.

## 1 Introduction

The interplay between physical system symmetry and energy band topology has been of great interest in topological physics since the discovery of the quantum Hall effect [1, 2]. Various symmetries, such as time-reversal symmetry, particle-hole symmetry, point group symmetry, and crystal space group symmetry, have been examined and may give rise to exotic topological phases, highlighting the crucial role of symmetry in topological classifications and topological band characterization [3–15]. Recently, the exploration of projectively represented space group (PRSG) symmetries has led to new discoveries in topological band physics [16–20]. Proposals have also been made to observe the effects of PRSG symmetries in PT-symmetric artificial phononic crystals [21].

It is well known that the presence of gauge degree of freedom can lead to a projective representation of the crystal symmetries. Notable examples include the creation of a new topological phase that features two Möbius twisted edge bands. These Möbius edge bands add to the richness of topological states and open up new avenues of research in topological physics [16]. Recently, topological phenomena based on PRSG symmetries and Möbius edge bands have been observed in both 2D and 3D acoustic crystals, utilizing the flexibility of coupling manipulation of acoustic waves between neighboring lattice sites [19, 20]. Most recently, due to the intrinsic multiorbitals in photonic waveguides, gauge fields are also incorporated into photonic waveguide arrays in square lattice. This has led to the prediction of characteristic Möbius twisted edge bands [22]. In Ref. [22], the application of the *s* and *d* orbitals is significant to build both “negative” and “positive” couplings between the fundamental optical modes (monopole and quadrupole) in the individual waveguides. We note that the synthetic gauge flux must be defined in the same direction for all the plaquettes in arrays with such configuration, quite similar to its acoustic counterparts [19, 20]. As a matter of fact, all these recently reported systems are essentially created by incorporating concepts from atomic physics and plasmonics, and feature an artificial gauge field [23–25]. In accord to the expectation from the tight binding approximation, a key feature is that the Möbius bands origins from the four-fold Dirac point at momentum **M** that is ensured by the projective symmetry.

**Figure 1:**
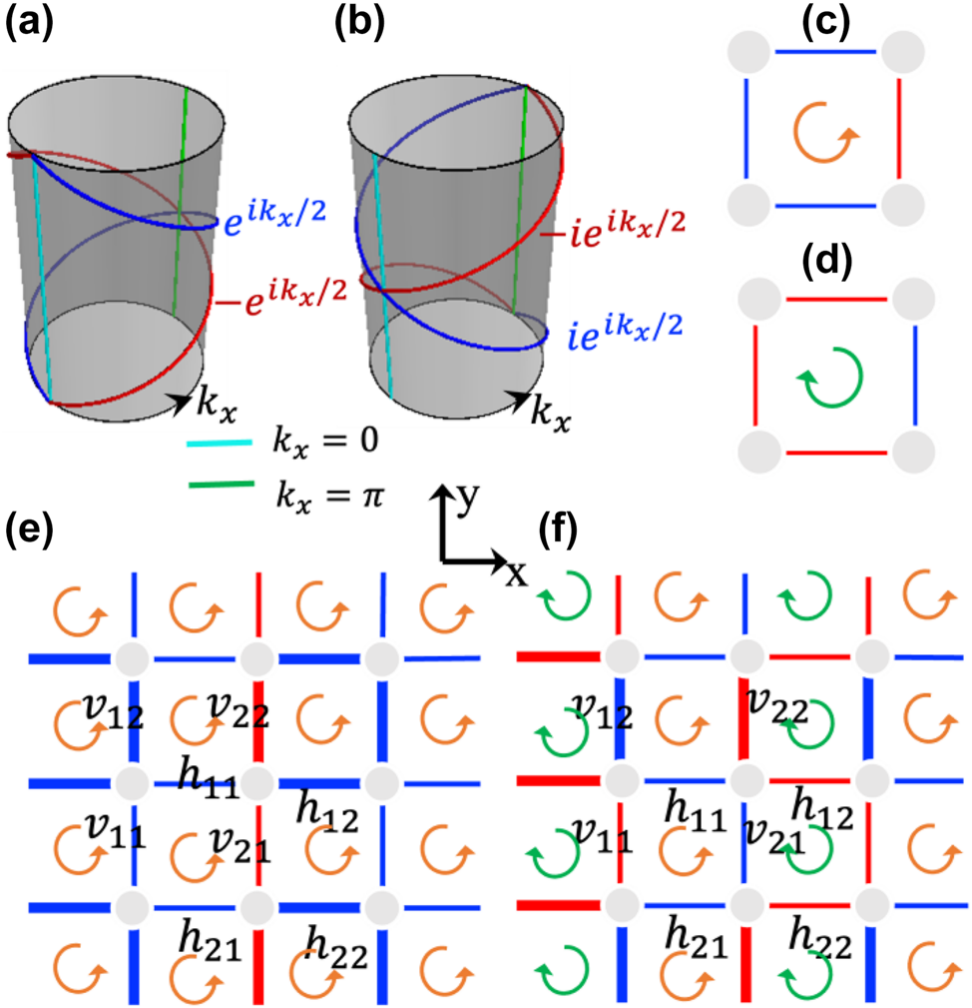
Illustration of the rectangular lattice models with *π* flux per plaquette. (a) And (b) schematic of the two Möbius twisted-band crossing at time-reversal symmetry invariant momenta *k*_*x*_ = 0, *π*, respectively. (c) And (d) schematic representation of two equivalent *π* gauge patterns under $Z_{2}$ gauge field: *π* and −*π* flux per plaquette. (e) Type-**I** gauge configuration where the hopping amplitudes along the *y* direction alternate in sign among the columns. (f) Type-**II** gauge configuration where the hopping amplitudes between adjacent bonds along both the *x* and *y* directions alternate in sign. The red (blue) bonds denote negative (positive) hopping amplitudes. The anti-clockwise and clockwise arrows indicate the *π* and −*π* gauge flux, respectively.

Figure 1a briefly shows the situation in both the acoustic and photonic systems mentioned above. Noticeably the twisted Möbius bands cross at *k*_*x*_ = *π* and are respectively characterized by projective translation operator eigenvalues $\pm {\text{e}}^{\text{i}k_{x}/2}$. However, Figure 1c and d show that the gauge flux can actually be defined as *π* or −*π* in hopping amplitude configurations (++ + −) or (−−− +), respectively. The corresponding lattice model is schematically depicted by Figure 1e. Apparently all the gauge flux is defined along the same direction (e.g., +*z*), and applies to both the constitute unit of Figure 1c and d. Here in this work, we introduce a different group of photonic mode coupling configuration that features alternating gauge flux *π* and −*π*, as shown in Figure 1f and show that it can be implemented in a photonic waveguide array. In such new cases, the flux pattern becomes staggering along one particular direction, for which the Möbius twisted bands can be tailored to cross at *k*_*x*_ = 0, as schematically shown in Figure 1b. The rotation of the Möbius twisted bands along parallel momentum (here *k*_*x*_) suggest the phase variation of the projective translation operator eigenvalues. That could be of relevance in beam steering control when exciting the edge bands. Remarkably, it is shown that such Möbius twisted bands can be realized by *s* and *p* orbitals couplings between the fundamental optical monopole and dipole modes. We provide concrete designs and waveguide geometries for possible observation of such edge bands. Mode coupling dynamic calculation of wave propagation inside the waveguide arrays is also presented, verifying the theoretical prediction.

## 2 Results

### 2.1 Theoretical model

After setting up the gauge field configurations in the 2D coupling lattices, one immediately observes that for the type-**I** configuration shown in Figure 1e, the primitive translation L_*y*_ is preserved, whereas primitive translation L_*x*_ is not preserved. While for the type-**II** configuration sketched in Figure 1f, both L_*x*_ and L_*y*_ are not preserved. To recover the original gauge pattern, an additional gauge transformation *G* must be incorporated [16]. Namely, under a gauge field, the proper primitive translation operator along the *x*/*y* direction is changed to L_*x*/*y*_ = *G*_*x*/*y*_L_*x*/*y*_. Here, for the type-**I** gauge configuration, the transformation *G*_*x*_ is associated with *G*_1_ where sites at odd (even) rows are multiplied with a *π* (0) phase, and the transformation *G*_*y*_ remains a unit transformation that leaves the original primitive translation unchanged. Whereas for the type-**II** configuration, *G*_*y*_ corresponds to *G*_1_, and *G*_*x*_ corresponds to *G*_2_, whereby sites located at diagonal (off-diagonal) positions undergo multiplication with a *π* (0) phase. Then, under the $Z_{2}$ gauge field, the anti-commutation relation between the rebuilt primitive translations is satisfied, i.e.,

(1) $$\left\{L_{x},L_{y}\right\}=0.$$

A consequence for the type-**I** $Z_{2}$ PRSG [see Figure 1e] is that each energy band is two-fold degenerated for a generic momentum **k** and a four-fold degenerate Dirac point appears at the high symmetry point *M* = (*π*, *π*) of the Brillouin zone (BZ) [19, 20]. In sharp contrast, for the type-**II** $Z_{2}$ PRSG [see Figure 1f], the eigenvalues of the translation operators, L_*x*_ and L_*y*_, are $\pm i{\text{e}}^{\text{i}k_{x}/2}$ and $\pm i{\text{e}}^{\text{i}k_{y}/2}$, respectively (see more in Supplementary Material Section 1). This is a significant hallmark of the Möbius insulator, wherein 4*π* periodicity is implied. Together with the time reversal symmetry (*T*) at the BZ center Γ = (0, 0), the dual eigenvalues are complex conjugated, contributing to Möbius twisted bands crossing at *k*_*x*/*y*_ = 0. Note that the twofold degeneracy at every **k** can also be understood by the projective PT symmetry, i.e., ${\left(PT\right)}^{2}=-1$ with $P={\text{G}}_{2}P$ (see Supplementary Material Section 2) [17, 18, 21].

To further elucidate the effect of the gauge field and the $Z_{2}$ PRSG, the general model Hamiltonian can be written as

(2) $$\begin{array}{ll} H\left(k\right)= & {\;t}_{x2}^{-}\sin k_{x}\Gamma_{1}-t_{y2}^{+}\sin k_{y}\Gamma_{2}+\left(t_{y1}^{+}+t_{y2}^{+}\cos k_{y}\right)\Gamma_{3}\\ & +\left(t_{x1}^{-}+t_{x2}^{-}\cos k_{x}\right)\Gamma_{4}+\left(t_{x1}^{+}+t_{x2}^{+}\cos k_{x}\right)i\Gamma_{1}\Gamma_{5}\\ & -\left(t_{y1}^{-}+t_{y2}^{-}\cos k_{y}\right)i\Gamma_{2}\Gamma_{5}-t_{y2}^{+}\sin k_{y}i\Gamma_{3}\Gamma_{5}\\ & +t_{x2}^{+}\sin k_{x}i\Gamma_{4}\Gamma_{5} \end{array}$$

where $t_{x1}^{\pm }=\left(h_{11}\pm h_{21}\right)/2$, $t_{x2}^{\pm }=\left(h_{12}\pm h_{22}\right)/2$, $t_{y1}^{\pm }=\left(v_{11}\pm v_{21}\right)/2$, $t_{y2}^{\pm }=\left(v_{12}\pm v_{22}\right)/2$, Γ_*μ*_ with *μ* = 1, 2, …, 5 are the 4 × 4 Dirac matrices: Γ_1_ = *τ*_3_ ⊗ *σ*_2_, Γ_2_ = *τ*_2_ ⊗ *σ*_0_, Γ_3_ = *τ*_1_ ⊗ *σ*_0_, Γ_4_ = *τ*_3_ ⊗ *σ*_1_, Γ_5_ = *τ*_3_ ⊗ *σ*_3_, with *τ* and *σ* being two sets of the Pauli matrices. The real hopping amplitudes *h*(*v*)_11_, *h*(*v*)_12_, *h*(*v*)_21_, and *h*(*v*)_22_ are indicated in Figure 1e and f. Under the $Z_{2}$ PRSG, the Hamiltonian for the type-**I** gauge configuration schematically shown in Figure 1e is reduced to $H_{\text{I}}\left(k\right)=t\left(1+cosk_{x}\right)i\Gamma_{1}\Gamma_{5}-t\left(1+\cos k_{y}\right)i\Gamma_{2}\Gamma_{5}-t\sin k_{y}i\Gamma_{3}\Gamma_{5}+t\sin k_{x}i\Gamma_{4}\Gamma_{5}$, with *t*the strength of all the equal hopping amplitudes, i.e., $|h_{11}\left|=\left|h_{12}\right|=|h_{21}\right|=\left|h_{22}\right|=|v_{11}|=\left|v_{12}\right|=|v_{21}|=\left|v_{22}\right|=t$.

**Figure 2:**
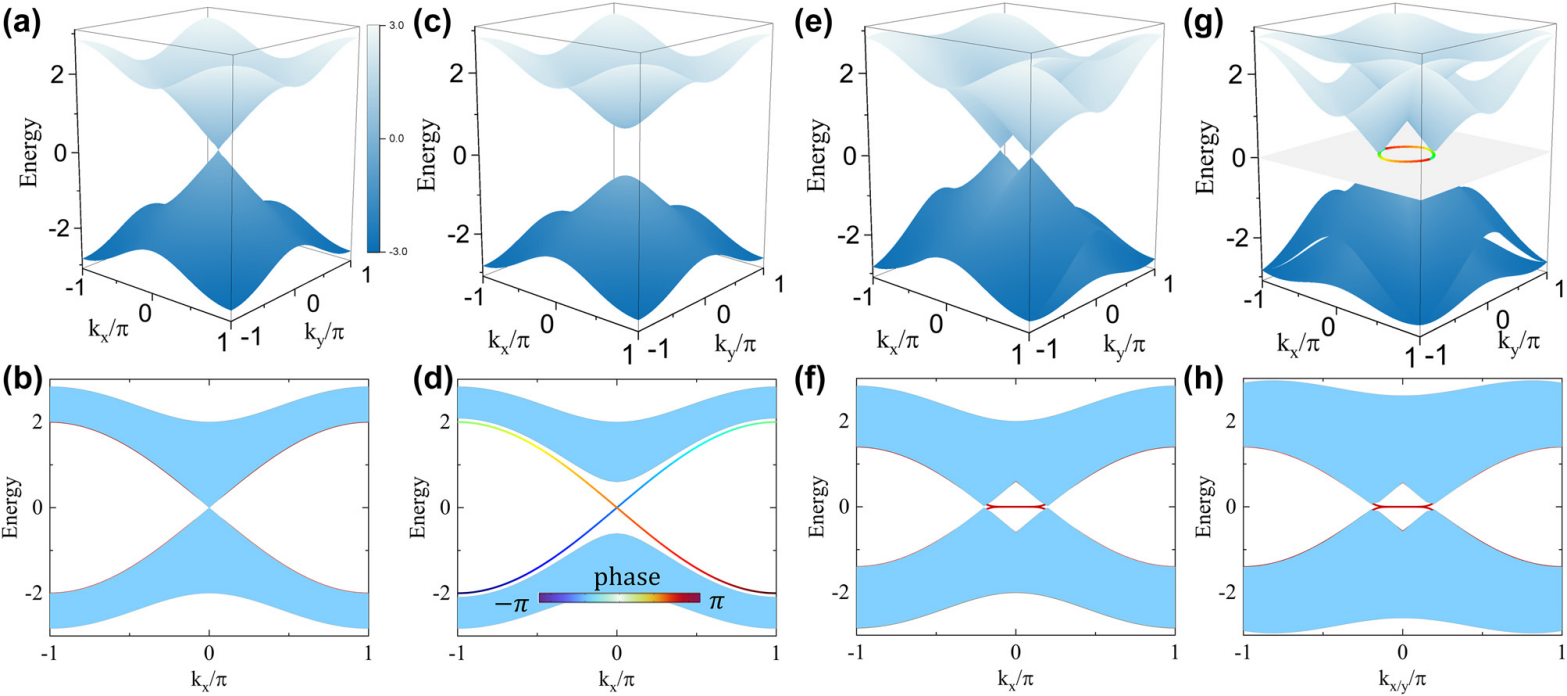
Band structure and edge bands. The band structure of the Hamiltonian (a) $H_{\text{II}}\left(k\right)$, (c) $H_{\text{III}}\left(k\right)$, (e) $H_{\text{IV}}\left(k\right)$ and (g) $H_{\text{V}}\left(k\right)$. The corresponding bulk bands are represented by blue curves while the edge bands are shown in red. The open edges in the *y* direction are illustrated in (b), (d), (f), and (h), respectively. The color map in (d) shows the phase evolution of the eigenvalues in the Möbius-twist edge bands. In (g), the bulk bands are for a specific case *θ* = *π*/4 of the dimerization ($|h_{11}|=\left|h_{22}\right|=t+t^{\prime }\sin\theta$, $|h_{12}|=\left|h_{21}\right|=t-t^{\prime }\sin\theta$, $|v_{11}|=\left|v_{22}\right|=t+t^{\prime }\cos\theta$, $|v_{12}|=\left|v_{21}\right|=t-t^{\prime }\cos\theta$). The colored ring indicates the trajectory of the *θ*-dependent nodal point splitting.

For the type-**II** gauge configuration [Figure 1f], the Hamiltonian becomes different to the Hamiltonian $H_{\text{I}}\left(k\right)$ whose four-fold Dirac point locates at (*k*_*x*_, *k*_*y*_) = (*π*, *π*) [19, 20], a four-fold degenerate point appears at (*k*_*x*_, *k*_*y*_) = (0, 0) for $H_{\text{II}}\left(k\right)$ as shown in Figure 2a. This agrees with the above theoretical analysis and represents a case of Dirac semimetal. The projected band for the corresponding finite-sized structure (e.g., truncated in the *y* direction) is shown in Figure 2b which clearly characterizes the gapless nodal point.

(3) $$\begin{array}{ll} H_{\text{II}}\left(k\right) & =t\left(1-cosk_{x}\right)i\Gamma_{1}\Gamma_{5}+t\left(1-\cos k_{y}\right)i\Gamma_{2}\Gamma_{5}\\ & \;-tsink_{y}i\Gamma_{3}\Gamma_{5}-tsink_{x}i\Gamma_{4}\Gamma_{5}\cdot \end{array}$$

Initiated with the four-fold Dirac semimetal, it is noted that selectively breaking the primitive translation L_*x*_ and/or L_*y*_ leads to a series of topological phase transition. For example, simultaneously breaking L_*x*_ and L_*y*_ yields the nontrivial quadrupole topological insulator. This has been demonstrated by setting |*v*_11_| < |*v*_12_|, |*v*_21_| < |*v*_22_|, |*h*_11_| < |*h*_12_| and $\left|h_{21}\right|<\left|h_{22}\right|$ to dimerize the intra- and inter-hopping intensity along both *x* and *y* directions [26–28]. On the other hand, breaking the primitive translation by dimerization of the hopping along only one direction would also enforce the bulk gap opening and give rise to novel topological physics. In the following, we focus on the type-**II** gauge configuration which could be realized in topological photonic Möbius waveguides as we show in the proceding section. The dimerization along *y*-axis is one of the simplest ways to achieve the desired symmetry breaking. In this way, the symmetry-constrained lattice model reads

(4) $$\begin{array}{ll} H_{\text{III}}\left(k\right) & =t\left(1-cosk_{x}\right)i\Gamma_{1}\Gamma_{5}+\left(t_{1}-t_{2}\cos k_{y}\right)i\Gamma_{2}\Gamma_{5}\\ & \;-t_{2}\sin k_{y}i\Gamma_{3}\Gamma_{5}-tsink_{x}i\Gamma_{4}\Gamma_{5}, \end{array}$$

where the strength of *v*_11_ and *v*_21_ (*v*_21_ and *v*_22_) is denoted as *t*_1_ (*t*_2_). The dimerization is introduced by setting *t*_1_ ≠ *t*_2_ which breaks the primitive symmetry L_*y*_, while L_*x*_ is still preserved in this case. Its band structure is shown in Figure 2c which becomes gapped. In addition, the model has a sublattice symmetry *S* characterized by Γ_5_, which anti-commutes with L_*x*_, i.e., $\left\{\hat{S},{\hat{L}}_{x}\right\}=0$. In the eigenspace of L_*x*_, the Hamiltonian $H_{\mathrm{III}}\left(k\right)$ can be block diagonalized on account of its sublattice symmetry

(5) $$H^{\prime }\left(k\right)=\left[\begin{array}{cc} h_{1}\left(k\right) & 0\\ 0 & h_{2}\left(k\right) \end{array}\right],$$

where

(6) $$h_{1,2}\left(k\right)=\left[\begin{array}{cc} 0 & q^{*}\left(k_{y}\right)\\ q\left(k_{y}\right) & 0 \end{array}\right]\mp m\left(k_{x}\right)\sigma_{3},$$

with $q\left(k_{y}\right)=t_{1}-t_{2}{\text{e}}^{\text{i}k_{y}}$ and $m\left(k_{x}\right)=2t\sin\left(k_{x}/2\right)$. Here, the first term in Equation (6) is nothing but the standard Su–Schrieffer–Heeger (SSH) model and the second term is a mass term. Note that for *t*_1_ < *t*_2_ the SSH model have topological nontrivial phase [29, 30]. Particularly, for *k*_*x*_ = 0, the system is decoupled to a pair of SSH models, which feature a pair of zero modes for an edge perpendicular to the *y* axis. Furthermore, there appear two Möbius-twist edge bands possessing opposite L_*x*_ eigenvalues $\ell_{\pm }=\pm i{\text{e}}^{\text{i}k_{x}/2}$ which have a period of 4*π* and are inverted after wrapping around the BZ once, as shown in Figure 2d [corresponding to the case in Figure 1(b)]. Interestingly, we note that this case corresponds to the topological Möbius insulator that has been discovered in acoustics [19, 20]. The $Z_{2}$ topological invariant is given by [16]

(7) $$v=\frac{1}{2\pi }\int_{\left[0,2\pi \right]\times S^{1}}Fd^{2}k+\frac{1}{\pi }\gamma \left(0\right)\;\mathrm{mod}\;2,$$

where $F=\nabla_{k}A$ is the Berry curvature with Berry connection $A\left(k\right)=\left\langle \psi_{-}|\text{i}\partial_{k}|\psi_{-}\right\rangle$ for the valence bands of *h*_1_ featured by eigenstate $\left.|\psi_{-}\right\rangle$, and $\gamma \left(k_{x}\right)$ is the Berry phase for subsystem *h*_1_(*k*_***x***_, *k*_*y*_) with fixed *k*_*x*_. A nontrivial *v* indicates a Möbius topological insulator, with Möbius-twist edge bands at any L_*x*_-invariant edge [26] (see more in Supplementary Material Section 3).

An alternating dimerization along the *y*-axis (i.e., $\left|v_{11}\right|=\left|v_{22}\right|=t_{1}<t_{2}=\left|v_{12}\right|=\left|v_{21}\right|$) would simultaneously breaks the primitive translation L_*x*_ and L_*y*_, yielding another distinct topological phase transition. For that, the Hamiltonian of the lattice model is then changed to

(8) $$\begin{array}{ll} H_{\text{IV}}\left(k\right) & =-\frac{t_{1}-t_{2}}{2}sink_{y}\Gamma_{2}-\frac{t_{1}-t_{2}}{2}\left(1+\cos k_{y}\right)\Gamma_{3}\\ & \;+t\left(1-\cos k_{x}\right)i\Gamma_{1}\Gamma_{5}+\frac{t_{1}+t_{2}}{2}\left(1-\cos k_{y}\right)\\ & \;\times i\Gamma_{2}\Gamma_{5}-\frac{t_{1}+t_{2}}{2}\sin k_{y}i\Gamma_{3}\Gamma_{5}-t\sin k_{x}i\Gamma_{4}\Gamma_{5}. \end{array}$$

In this case, the four-fold degenerate Dirac point splits into two nodal points with a two-fold degeneracy along the line *k*_*y*_ = 0, as shown in Figure 2e. This is in similar to the creation of a Weyl point pair from a four-fold degenerate Dirac point [31]. The topological charge can be determined by calculating the winding number around a circle *C* surrounding the nodal point: $w={\left(1/4\pi i\right)\oint }_{C}dk\cdot \text{tr}\Gamma_{5}H^{-1}\left(k\right)\nabla H\left(k\right)$ [19].

Figure 2e shows the complete band structure and Figure 2f shows that a flat edge band connecting the projections of the two nodal points, and the 2D counterpart of the Fermi-arc connecting the projection of the Weyl points, can be observed due to the nontrivial topological charge [32, 33]. To further break the hopping terms along the *x* direction, the position of the paired nodal points can be modulated in the full quadrants of the momentum space, instead of just being along the high-symmetric lines. To exemplify that, the dimerization along both the *x* direction ($|h_{11}|=\left|h_{22}\right|=t+t^{\prime }\sin\theta$, $|h_{12}|=\left|h_{21}\right|=t-t^{\prime }\sin\theta$) and the *y* direction ($|v_{11}|=\left|v_{22}\right|=t+t^{\prime }\cos\theta$, $|v_{12}|=\left|v_{21}\right|=t-t^{\prime }\cos\theta$) is applied and the Hamiltonian becomes

$$\begin{array}{ll} H_{\text{V}}\left(k\right) & =t^{\prime }sin\theta \sin k_{x}\Gamma_{1}+t^{\prime }\cos\theta \sin k_{y}\Gamma_{2}-t^{\prime }\cos\theta \\ & \;\times \;\left(1+\cos k_{y}\right)\Gamma_{3} \end{array}$$

$$\begin{array}{ll} & +t^{\prime }\sin\theta \left(1+\cos k_{x}\right)\Gamma_{4}+t\left(1-\cos k_{x}\right)i\Gamma_{1}\Gamma_{5}\\ & +t\left(1-\cos k_{y}\right)i\Gamma_{2}\Gamma_{5} \end{array}$$

(9) $$\;-\;t\sin k_{y}i\Gamma_{3}\Gamma_{5}-t\sin k_{x}i\Gamma_{4}\Gamma_{5}.$$

In this case, the paired nodal points are *θ*-dependent and behave as the colored ring shown in Figure 2g. Particularly for *θ* = *π*/4, Figure 2g shows that the bulk bands exhibit the linear crossing in the vicinity of the paired nodal points which fall on the momentum line Γ*M*. Due to the nontrivial topological charge, an edge band appears for an open edge either in the *x* or in the *y* direction, as shown in Figure 2h.

### 2.2 Full wave simulation and coupled model theory

**Figure 3:**
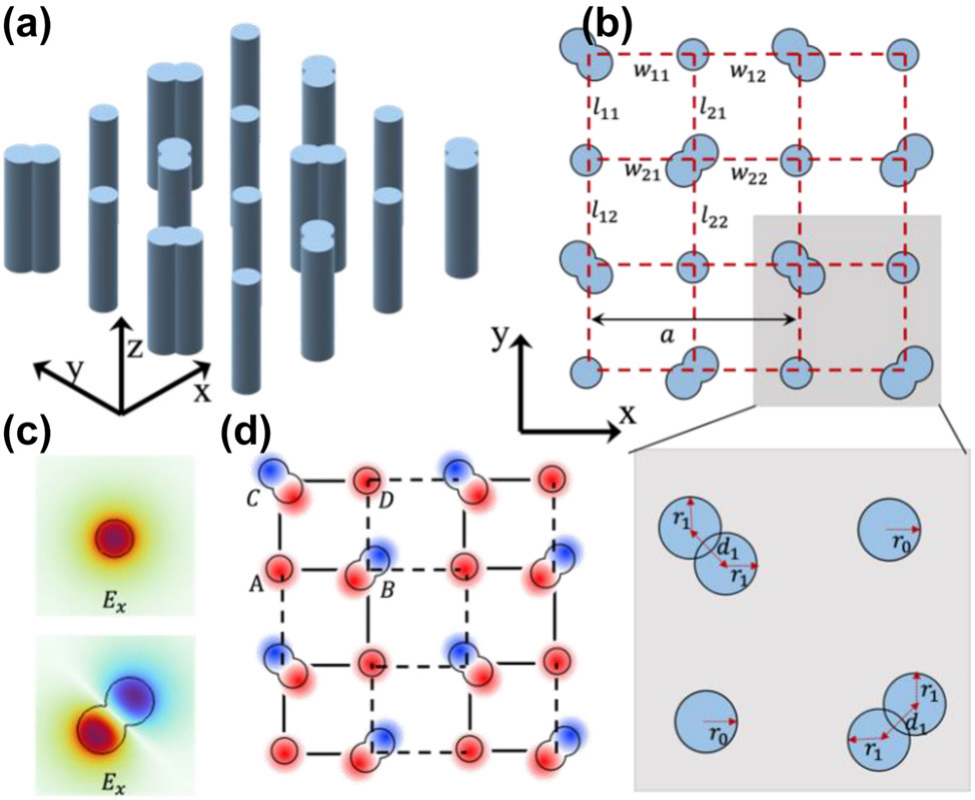
The designed photonic waveguide structures. (a) 3D schematic structure of the topological photonic Möbius waveguide array and (b) the cross-section view. (c) Fundamental mode (at *λ* = 760 nm) pattern Re(*E*_*x*_) of the individual circular waveguide and the peanut waveguide, resembling the *s* and *p* orbitals, respectively. (d) Illustration of the coupling behavior among the *s* and *p* orbitals in the waveguide lattices. The radius of the isolated circular waveguide is *r*_0_ = 0.4625 μm. The radius and the center-to-center distances of the constituent circular parts forming a peanut waveguide are *r*_1_ = 0.6 μm and *d*_1_ = 1 μm, respectively. The zoom-in unit cell shaded in gray in (b) is shown at the bottom panel. The lattice constant is *a* = 6 μm. The distances between the waveguides in the *x*(*y*) directions, *w*_11_, *w*_12_, *w*_21_, and *w*_22_ (*l*_11_, *l*_12_, *l*_21_, and *l*_22_), can be varied to modulate the intra- and inter-hopping amplitude. The refractive index of the waveguide and the host are *n*_*co*_ = 1.59, and *n*_*cl*_ = 1.54, respectively. Note that solid and dashed line labeled in (d) indicates the positive and negative coupling constant due to the directional arrangement of the *p* orbital.

In the previous section, we demonstrated the richness of topological phases and the accompanying edge modes. In this section, we move on to showcase the implementation and potential realization of these theoretically predicted phenomena in 3D photonic waveguide arrays that have the type-**II** gauge field. Figure 3a illustrates the geometry of the waveguide structure, composed of high-refractive index waveguides with circular and peanut-like cross-sections. Figure 3b displays a cross-sectional slice. It is important to note that the peanut-like waveguides are constructed by overlapping two circular waveguides. The radii and center-to-center distance can be adjusted to support eigenmodes with the same propagation constant for the fundamental mode in the individual circular waveguide (see Section 4 of the Supplementary Material). The peanut waveguides at opposite diagonal sites of a unit cell are tilted with respect to the *y*-axis by ± 45°, respectively. Unlike in acoustic lattices discussed in Refs. [19, 20], in the coupled photonic waveguide system, the coupling strength and sign are determined by the overlap of the decoupled base states. The coupling between the *i*th state $\left.\left|E_{i}\right.\right\rangle$ and the *j*th state $\left.|E_{j}\right\rangle$ is proportional to $\left\langle E_{i}|E_{j}\right\rangle$ [34, 35]. Figure 3c shows the fundamental modal electric field *E*_*x*_ (at *λ* = 760 nm) of the decoupled circular and peanut waveguides, in the form of monopole (*s* orbital) and dipole (*p* orbital), respectively. The results were obtained from the numerical simulations using the finite element method (FEM) in COMSOL Multiphysics^®^ 5.0. Clearly, *p* orbitals have ‘negative parts’ which contribute to the negative coupling. In the waveguide lattices shown in Figure 3b, the nearest-neighboring modal coupling can be represented by the constituent *s* and *p* orbitals, as shown in Figure 3d. Then, the positive (negative) couplings in the waveguide lattices are labeled by the solid (dashed) bonds. The coupling strength (the absolute value of the coupling coefficients) can be controlled by the site distance. The diagonal site couplings have been ignored in this work as they are relatively small (see Supplementary Material S6). By using the *s* and *p* orbitals, the *π* flux threading a plaquette is implemented [25]. Note that using *s* and *d* orbitals can implement the type-I gauge configuration shown in Figure 1a and e. Here in our case, each waveguide supports a pair of orbitals: *s* (*s*_1_ and *s*_2_) and *p* (*p*_1_ and *p*_2_) (see Supplementary Material Section 4). The coupling in the waveguide arrays can be in the form of *s*_1_ − *p*_2_ − *p*_1_ − *s*_1_ and *s*_2_ − *p*_1_ − *p*_2_ − *s*_2_, following the order *A*− *B* − *C* − *D* in Figure 3d.

**Figure 4:**
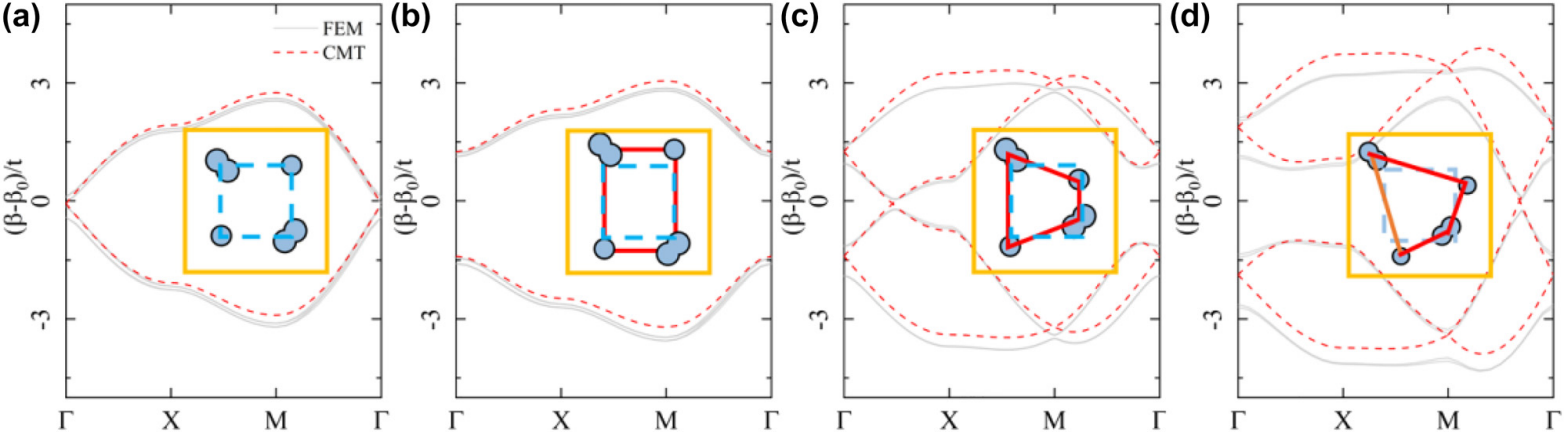
Comparison between the band structures calculated using the full-wave numerical method (gray solid line) and the CMT (red dashed line) for: (a) the Dirac semimetal case with *w*_11_ = *w*_12_ = *w*_21_ = *w*_22_ = *l*_11_ = *l*_12_ = *l*_21_ = *l*_22_ = *a*/2, (b) the Möbius insulator case with *w*_11_ = *w*_12_ = *w*_21_ = *w*_22_ = *a*/2, *l*_11_ = *l*_21_ = *a*/2 + 0.03*a*, and *l*_12_ = *l*_22_ = *a*/2 − 0.03*a*, (c) the Weyl-like semimetal case with *w*_11_ = *w*_12_ = *w*_21_ = *w*_22_ = *a*/2, *l*_11_ = *l*_22_ = *a*/2 + 0.03*a*, and *l*_12_ = *l*_21_ = *a*/2 − 0.03*a*, and (d) the Weyl-like semimetal case with *w*_11_ = *w*_22_ = *a*/2 + 0.03*a*, and *w*_12_ = *w*_21_ = *a*/2 − 0.03*a*, *l*_11_ = *l*_22_ = *a*/2 + 0.03*a*, and *l*_12_ = *l*_21_ = *a*/2 − 0.03*a*. We note that a comprehensive phase diagram with respect to the geometric dimerization can be found in the Supplementary Material Table 1 (Section 5).

The propagating waveguide mode in the waveguide array can be strongly coupled and described by the Schrödinger-like wave equation. Within the coupled mode theory (CMT) the steady state equation reads [36, 37]:

(10) $$i\frac{\partial E\left(r,z\right)}{\partial z}=\hat{H}E\left(r,z\right).$$

where *E*(**r**, *z*) is the transverse electric field at the propagation distance *z*. The components of $\hat{H}$ can be obtained by the overlapping integral of the fundamental modal fields based on the CMT. Due to the consistency between the Hamiltonian $\hat{\text{H}}$ obtained by the CMT and H(k) described by Equation (2), a couple of topological phase transitions of the propagating modes are expected, which shall be intrinsic to these waveguide arrays (see Supplementary Material Section 5). Note that for the specific design in Figure 3, when there is no hopping/coupling dimerization (i.e., *l*_11_ = *l*_12_ = *l*_21_ = *l*_22_ = *w*_11_ = *w*_12_ = *w*_21_ = *w*_22_ = *a*/2, seen in the inset of Figure 4a), the propagation constant and the nearest-neighboring coupling between the waveguides are *β*_0_ = 1.5514*k*_0_ and *t* = 0.00027*k*_0_, respectively, where *k*_0_ is the wavenumber in free space at the working frequency (*λ* = 760 nm) (see Supplementary Material Section 6). Figure 4a shows that the bulk bands obtained by the CMT are in good agreement with the full-wave numerical data from the FEM. Clearly, the bulk bands are two-fold degenerated, and a four-fold Dirac point appears at Γ point. To break the primitive translation symmetry L_*y*_, the dimerization in the waveguide arrays can be realized by altering the center-to-center distances of the individual waveguides, i.e., by setting *l*_11_ = *l*_21_ = *a*/2 + *h* and *l*_12_ = *l*_22_ = *a*/2 − *h*.

**Figure 5:**
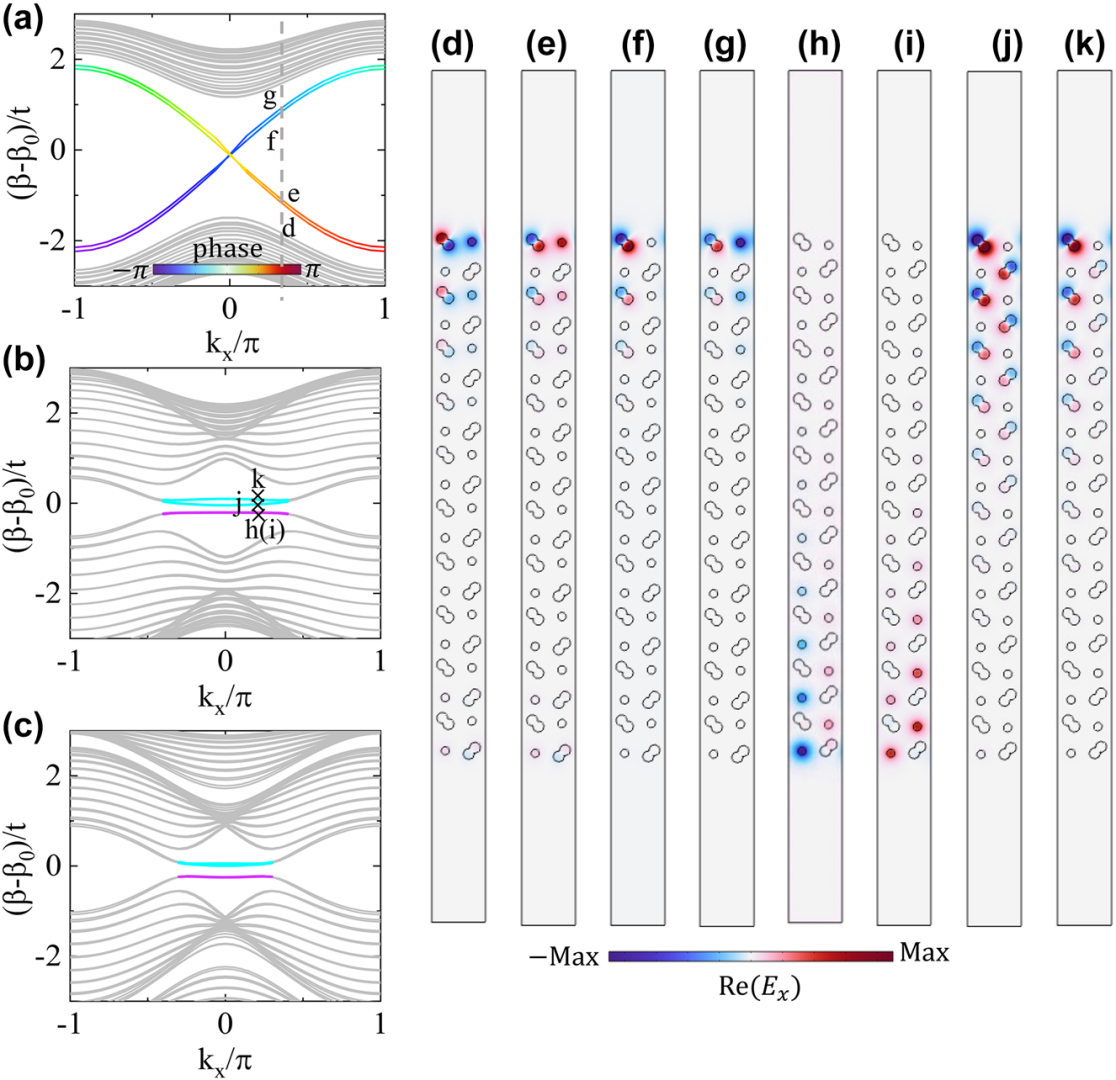
Simulated dispersions for supercell structures with open boundary condition in the *y* direction and the periodic boundary condition in the *x* direction. (a) Möbius insulator for *w*_11_ = *w*_12_ = *w*_21_ = *w*_22_ = *a*/2, *l*_11_ = *l*_21_ = *a*/2 + 0.03*a*, and *l*_12_ = *l*_22_ = *a*/2 − 0.03*a*, (b) Weyl-like semimetal phases for dimerization along the *y* direction: *l*_11_ = *l*_22_ = *a*/2 + 0.03*a* and *l*_12_ = *l*_21_ = *a*/2 − 0.03*a*, (c) Weyl-like semimetal phase for dimerization along both axis: *l*_11_ = *l*_22_ = *a*/2 + 0.03*a*, *l*_12_ = *l*_21_ = *a*/2 − 0.03*a*, *w*_11_ = *w*_22_ = *a*/2 + 0.03*a*, and *w*_12_ = *w*_21_ = *a*/2 − 0.03*a*. The eigenmode patterns at *k*_*x*_ = 0.2*π* as labeled in (a) and (b) are shown for the Mobius-twist edge states (d–g) and the Weyl-like edge states (h–k), respectively.

Firstly, we impose a staggered dimerization pattern by setting *h* = 0.03*a* (see the inset of Figure 4b). Since the primitive translation L_*y*_ is broken, a band gap is opened, as shown in Figure 4b. Here, the coupling along the *y*-axis obtained by the CMT are *t*_1_ = 0.00015*k*_0_ and *t*_2_ = 0.0005*k*_0_. In accordance with the tight-binding model Equation (4), the four-fold degenerate Dirac point becomes gapped, and two Möbius twisted topological edge bands in Figure 2d are expected to emerge. To observe these Möbius-twist edge states, an open boundary condition parallel to the *x*-axis is imposed on a finite structure truncated in the *y* direction. Figure 5a shows the band structures for a ‘ribbon-like’ structure with 10 periods in the *y* direction. It is seen that there appear four edge bands inside the band gap, with the edge states associated with L_*x*_ eigenvalues clearly indicated by the phase colorbar. As a hallmark of the Möbius topology, the phase of the eigenvalues exhibits a 4*π* periodicity. Since the composed waveguides intrinsically host two degenerate modes, two pairs of Möbius-twist edge states are observed at the upper boundary (waveguides in the top layer) of the ‘ribbon’ structure. The field patterns of the edge states at *k*_*x*_ = 0.2*π* are, respectively, shown in Figure 5d–g, and it is clear that these edge fields are primarily localized at the waveguides near the upper boundary, with different phases. This represents a key characteristic of the Möbius-twist edge bands. We note that actually there are also four Möbius edge bands for the lower boundary of the ‘ribbon’ structure. These edge band states residue in the lower boundary (waveguides in the lower layer) degenerate with those in the upper boundary (details are not shown).

Secondly, to build different dimerization pattern, *h* = 0.03*a* and *h* = −0.03*a* are applied to the two neighboring columns of the unit cell simultaneously. This results in the 2D Weyl-like semimetal phase defined by Equation (8) and Figure 2e. In this case, the four-fold degenerate Dirac point is transformed into a pair of twofold nodal points along the momentum line Γ − *X*. The results are shown in Figure 4c. Again, the CMT results agree well with the numerical simulations. In analogy to Weyl point and Fermi arc [18, 31], [32], [33], these 2D nodal points host nontrivial topological charge, which leads to the edge band connecting the projections of the nodal points. The results in the waveguide arrays are shown in Figure 5b. It is seen that four edge bands (two degenerate ones marked by the purple curves and two isolated ones by the cyan curves) connecting the projections of the nodal points. As a matter of fact, the next-nearest-neighbor coupling between the off-diagonal sites cannot be completely neglected which gives rise to a small bulk gap between them (see Section 7 in Supplementary Material). Figure 5h–k shows the eigenmode patterns for these edge modes. Different to the Mobius-twist edge state, these Fermi-arc like edge states localized at the lower and the upper boundaries of the waveguide array are mainly confined at the circular waveguide and the peanut waveguide, respectively. Besides, a slight deviation of the edge bands is due to a slight mismatch between the fundamental mode propagation wavenumber *β*_0_ of the isolated circular and peanut waveguides.

Finally, to further break the intrinsic symmetries in the waveguide lattices, we set *w*_11_ = *w*_22_ = *a*/2 + 0.03*a*, *w*_12_ = *w*_21_ = *a*/2 − 0.03*a*, *l*_11_ = *l*_22_ = *a*/2 + 0.03*a*, and *l*_12_ = *l*_21_ = *a*/2 − 0.03*a* as shown in the inset of Figure 4d. In this case, only the time-reversal symmetry is preserved, and two nodal points appear on the line Γ*M*. This corresponds to the case of *θ* = *π*/4 of the model shown in Figure 2g. Figure 5c shows the projected band structures and the edge states, which is similar to the case of Figure 5b. Both cases are able to generate 2D Weyl-like phase. Certainly, similar phenomena can be observed for another configuration with arbitrary *θ* (results not shown here).

### 2.3 Coupled mode dynamics

**Figure 6:**
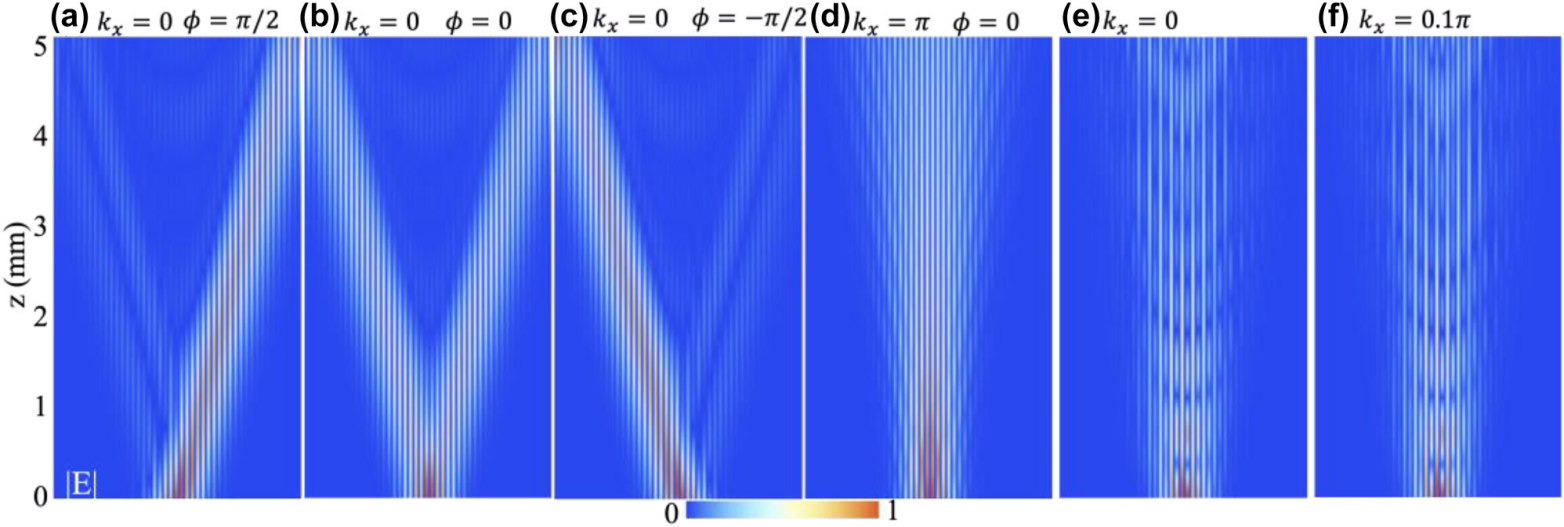
Wave dynamics inside the waveguide array excited by a incident Gaussian beam with various initial spatial phase difference, for (a)–(d) the Möbius twisted bands and (e)–(f) the Weyl-like semimetal flat bands: (a) *k*_*x*_ = 0, *ϕ* = *π*/2, (b) *k*_*x*_ = 0, *ϕ* = 0, (c) *k*_*x*_ = 0, *ϕ* = −*π*/2, (d) *k*_*x*_ = *π*, *ϕ* = 0, (e) *k*_*x*_ = 0, and (f) *k*_*x*_ = 0.1*π*.

We have shown that the Möbius insulator and Weyl-like semimetal are characterized by distinct topological edge bands. It is possible to show the linear crossing of the Möbius-twisted edge bands at *k* = 0 and the flat characteristic of the Fermi-arc-like edge band can be demonstrated with full-wave simulation. In view of the good agreements between the CMT and the FEM results and for the sake of simplicity, we provide a coupled mode dynamics calculation based on Equation (10). Specifically, we construct an array comprised of 3 × 23 unit cells (see Figure 3) and launch a Gaussian beam $P\left(x,\phi \right)=\exp\left(-{\left(x-x_{0}\right)}^{2}/\Delta^{2}\right)\exp\left(ik_{x}x+i\phi \right)$ impinging upon the top row waveguides to excite the edge state. Here *x* labels the waveguide position, *x*_0_ is the beam center and Δ the beam width. A phase difference *ϕ* = arg(*ℓ*_±_) − *π* was applied to the top layer waveguides within a unit cell to align each individual Möbius band with a fixed *k*_*x*_ [20]. Figure 6a–d show the results, illustrating four representative cases of beam dynamics for various combinations of *k*_*x*_ and *ϕ*. Due to the linear crossing and twist characteristics of the edge bands around *k*_*x*_ = 0, the propagating beams can be modulated to travel towards the right or left, or bifurcate, by adjusting the phase difference *ϕ* to *π*/2, 0, and −*π*/2, as depicted in Figure 6a–c. However, as shown in Figure 6d, when the launched beam momentum is set to *k*_*x*_ = *π*, the excited wave propagates almost along the *z* axis without obvious deflection. This clearly shows the *π* rotation of the twisting bands shown in Figure 1a and b. Moreover, due to the flatness of the Weyl-like semimetal edge band, the excited beam is restricted to propagation along the *z* axis, as demonstrated in Figure 6e and f.

## 3 Conclusions

Before we conclude, a few comments are in order. (1) The waveguide arrays can be fabricated using techniques such as femtosecond laser direct writing [36] and two-photon lithography [37]. However, in order to observe the excitation wave profile-dependent edge mode dynamic in the designed system shown in Figure 6, the length of the waveguide shall be roughly no less than 2 mm. This posts great challenges on fabrication stability since the cross section of the waveguide is at the order of half micrometer. (2) Our proposed geometry design readily allows for the observation of Möbius edge states, and it may also be possible to involve PT symmetry (let (*PT*)^2^ = −1), construct spinful edge states [21], or even have Weyl edge states in conjunction with them in waveguide arrays [38]. Exploring higher-dimensional cases may also be meaningful and significant [39]. (3) Moreover, new physical signatures resulting from projective symmetry algebras such as the shift of high-symmetry momenta, enforced nontrivial Zak phase, and spinless eight-fold nodal point theoretically predicted by Chen et al. [40] may be implemented and observed in plasmonic ellipsoidal nanoparticle arrays constructed in both 2D array and 3D array, since these plasmonic systems provide a flexible platform to engineering both the interaction strength and polarity (e.g., the amplitude and sign of coupling coefficients), at least within the dipole resonance approximation [41, 42].

In conclusion, we demonstrate for the first time the potential for observing topological phases protected by projective crystal symmetries in photonic waveguide arrays. Specifically, we introduce a novel type of gauge field configuration that can be realized in 3D waveguide arrays through the analogous photonic *s* and *p* orbitals and their couplings. Our findings reveal that the fundamental structure of the waveguide arrays enforces a four-fold degenerate Dirac point at the center of the Brillouin zone through two primitive transition symmetries. By introducing dimerization with a transverse shift of the waveguide position, the primitive transition symmetry is broken, leading to the emergence of a Möbius topological insulator and a Weyl-like semimetal.

## Supplementary Material

Supplementary Material Details
