# Supplementary material for: Möbius edge band and Weyl-like semimetal flat-band in topological photonic waveguide array by synthetic gauge flux
Source: Nanophotonics. 2023 Jul 14;12(17):3481–90. doi: 10.1515/nanoph-2023-0311 (PMC11501785; doi:10.1515/nanoph-2023-0311)
Supplement: Supplementary file 1 — Supplementary Material Details [file j_nanoph-2023-0311_suppl_001.pdf]

Supplementary Material

Möbius edge band and Weyl-like semimetal flat-band in topological photonic waveguide array by synthetic gauge flux

Zhenzhen Liu[1,2], Guochao Wei[1,2,3], Huizhou Wu[1,2], and Jun Jun Xiao[1,2,*]

[1]College of Electronic and Information Engineering, Harbin Institute of Technology (Shenzhen), Shenzhen 518055, China

[2]Shenzhen Engineering Laboratory of Aerospace Detection and Imaging, Harbin Institute of Technology (Shenzhen), Shenzhen 518055, China

[3]School of Mathematical and Physical Sciences, Wuhan Textile University, Wuhan 430200, China

[*]E-mail: eiexiao@hit.edu.cn

Contents

1. Primitive translation symmetry and the four-fold degenerate Dirac point 2
2. Projective PT symmetry 3
3. Möbius topological insulator 3
4. Eigenmode of the decoupled circular and peanut waveguides 4
5. The topological phase diagram with respect to the geometrical dimerization...........6
6. Coupled mode theory results for the waveguide array 6
7. The effect of the next-nearest-neighbor coupling for the Weyl Hamiltonian 8
8. The field profile for the various edge states...10
9. The eigenvalues of the projective translation symmetry 11

1. Primitive translation symmetry and the four-fold degenerate Dirac point

Under the gauge configuration shown in Figure 1 of the main text, the primitive translation operator along x and y is not manifestly preserved. To recover the original gauge pattern, an additional gauge transformation G must be incorporated which adds a π phase for the sites in odd rows. Namely, under a gauge field, the proper primitive translation operator along the x/y direction is changed to $\mathrm{L}_{x/y} = \mathrm{G}_{\mathrm{x/y}} L_{x/y}$, here G_{y} corresponds to the transformation G_1 for which the sites at odd (even) rows are multiplied with a π (0) phase [see Figure 1c of the main text], and G_{x} corresponds to the transformation G_2 shown in Figure 1d where sites at diagonal (off-diagonal) are multiplied with a π (0) phase. In this regard, under the gauge condition, the proper primitive translation operators L_y anti-commutes with L_x as [S1],

$$\{\mathrm{L}_x, \mathrm{L}_y\} = 0 \tag{1}$$

To consider the representations of the translation operators in momentum space, the square unit cell with four sites is selected. Then, L_x and L_y are represented in matrix form by

$$\hat{L}_{\mathrm{x}} = \tau_0 \otimes \begin{bmatrix} 0 & 1 \\ e^{ik_x} & 0 \end{bmatrix}, \tag{2}$$

$$\hat{L}_{\mathrm{y}} = \begin{bmatrix} 0 & 1 \\ e^{ik_y} & 0 \end{bmatrix} \otimes \sigma_0. \tag{3}$$

The gauge transformations G_1 and G_2 are represented by

$$\hat{\mathrm{G}}_1 = \tau_3 \otimes \sigma_0, \tag{4}$$

$$\hat{\mathrm{G}}_2 = \Gamma_5. \tag{5}$$

Hence, the proper translation operators are given by

$$\hat{\mathrm{L}}_{\mathrm{x}} = \hat{\mathrm{G}}_2 \hat{L}_{\mathrm{x}} = \tau_3 \otimes \begin{bmatrix} 0 & 1 \\ -e^{ik_x} & 0 \end{bmatrix}, \tag{6}$$

$$\hat{\mathrm{L}}_{\mathrm{y}} = \hat{\mathrm{G}}_1 \hat{L}_{\mathrm{y}} = \begin{bmatrix} 0 & 1 \\ -e^{ik_y} & 0 \end{bmatrix} \otimes \sigma_0. \tag{7}$$

This is consistent with the anti-commutation relation in Eq. (1).

Since $[\hat{\mathrm{L}}_{\mathrm{x}}, \mathcal{H}(\boldsymbol{k})] = 0$, the eigenstate $\psi(\boldsymbol{k})$ must simultaneously be the eigenstate of $\mathcal{H}(\boldsymbol{k})$ and $\hat{\mathrm{L}}_{\mathrm{x}}$. Namely, $\mathcal{H}(\boldsymbol{k})\psi(\boldsymbol{k}) = E(\boldsymbol{k})\psi(\boldsymbol{k})$, and $\hat{\mathrm{L}}_{\mathrm{x}}\psi(\boldsymbol{k}) = \pm i e^{ik_x/2}\psi(\boldsymbol{k})$. With regard to Eq. (1), $\hat{\mathrm{L}}_{\mathrm{x}}\hat{\mathrm{L}}_{\mathrm{y}}\psi(\boldsymbol{k}) = -\hat{\mathrm{L}}_{\mathrm{y}}\hat{\mathrm{L}}_{\mathrm{x}}\psi(\boldsymbol{k}) = \mp i e^{ik_x/2}\hat{\mathrm{L}}_{\mathrm{y}}\psi(\boldsymbol{k})$. Then, $\hat{\mathrm{L}}_{\mathrm{y}}\psi(\boldsymbol{k})$ is also an eigenstate of $\hat{\mathrm{L}}_{\mathrm{x}}$ but with the opposite eigenvalue $\mp i e^{ik_x/2}$. Therefore, $\psi(\boldsymbol{k})$ and

$\hat{\mathrm{L}}_{\mathrm{y}}\psi(\boldsymbol{k})$ are orthogonal. Moreover, together with $[\hat{\mathrm{L}}_{\mathrm{y}},\mathcal{H}(\boldsymbol{k})]=0$, $\hat{\mathrm{L}}_{\mathrm{y}}\psi(\boldsymbol{k})$ is also an eigenstate of $\mathcal{H}(\boldsymbol{k})$ with the same energy $E(\boldsymbol{k})$. Therefore, each energy bands are two-fold degenerated.

In addition to the primitive translation symmetries characterized by L_x and L_y, the inherent time-reversal symmetry is also preserved, which is represented by $\hat{\mathcal{T}}=\hat{\mathcal{K}}$, where $\hat{\mathcal{K}}$ denote the complex conjugation. Particularly, at momentum point $\Gamma=(0,0)$, the time-reversal symmetry is satisfied and the eigenvalues of $\hat{\mathrm{L}}_{\mathrm{x}}$ become $\pm i$. Therefore, the eigenstates with complex eigenvalues are degenerate, which gives rise to a fourfold degeneracy point.

2. Projective PT symmetry

In addition to the primitive translation symmetry along x and/or y directions, the space inversion symmetry (P) can also be projectively represented as $\mathcal{P}=G_1P$. P is represented by $\hat{\mathrm{P}}_1=\tau_1\otimes\sigma_1$. In this case, the time-reversal operator (T) is defined as the complex conjugate, i.e., $\hat{\mathrm{P}}_1=\hat{\mathcal{K}}$, then the projective PT symmetry satisfy $(\mathcal{P}T)^2=-1$.

3. Möbius topological insulator

Since $[\mathrm{L}_x,\mathcal{H}(\boldsymbol{k})]=0$, $\hat{\mathrm{L}}_{\mathrm{x}}$ can be diagonalized as

$$U^{\dagger}\hat{\mathrm{L}}_{\mathrm{x}}U=ie^{ik_x/2}\tau_3\otimes\sigma_3, \tag{8}$$

where

$$U(k_x)=\frac{1}{\sqrt{2}}\begin{bmatrix}0 & ie^{-\mathrm{i}k_x/4} & 0 & -ie^{-\mathrm{i}k_x/4}\\ 0 & e^{\mathrm{i}k_x/4} & 0 & e^{\mathrm{i}k_x/4}\\ -ie^{-\mathrm{i}k_x/4} & 0 & ie^{-\mathrm{i}k_x/4} & 0\\ e^{\mathrm{i}k_x/4} & 0 & e^{\mathrm{i}k_x/4} & 0\end{bmatrix}. \tag{9}$$

And the Hamiltonian can be decomposed into two off-diagonal blocks as

$$U^{\dagger}\mathcal{H}U=\begin{bmatrix}h_1(\boldsymbol{k}) & 0\\ 0 & h_2(\boldsymbol{k})\end{bmatrix}. \tag{10}$$

The sublattice symmetry S is transformed as

$$U^{\dagger}\,\hat{\mathcal{S}}U=\tau_1\otimes\sigma_3. \tag{11}$$

Hence, the sublattice symmetry requires that

$$\sigma_3h_1(\boldsymbol{k})\sigma_3=-h_1(\boldsymbol{k}), \tag{12}$$

$$\sigma_3h_2(\boldsymbol{k})\sigma_3=-h_2(\boldsymbol{k}). \tag{13}$$

Note that the projective nature of L_x, U is not periodic in k_x,

$$U(k_x + 2\pi) = V(k_x)U(k_x). \tag{14}$$

$V(k_x)$ is constantly represented as $V(k_x) = -i\tau_0 \otimes \sigma_3$. Consequently, $U^\dagger \mathcal{H} U$ is also not periodic in k_x, but satisfies the following relation

$$\sigma_3 h_{1,2}(k_x, k_y)\sigma_3 = -h_{1,2}(k_x + 2\pi, k_y). \tag{15}$$

Explicitly, for our model

$$h_{1,2}(\boldsymbol{k}) = \begin{bmatrix} 0 & q^*(k_y) \\ q(k_y) & 0 \end{bmatrix} \mp m(k_x)\sigma_3, \tag{16}$$

where $q(k_y) = t_1 - t_2 e^{ik_y}$, $m(k_x) = 2t\sin(k_x/2)$. The first term is nothing but the standard Su-Schrieffer-Heeger (SSH) model, and the second term is a mass term.

$$h_1(\mathbf{k}) = \begin{bmatrix} -2t\sin(\frac{k_x}{2}) & \mathrm{t}_1 - e^{-ik_y}\mathrm{t}_2 \\ t_1 - e^{ik_y}t_2 & 2t\sin(\frac{\mathrm{k_x}}{2}) \end{bmatrix}. \tag{17}$$

Assuming that the eigenstate for the valence band is $|\psi_-\rangle$, the Berry connection is defined as $\mathcal{A}_2 = \langle\psi_-|\mathrm{i}\,\partial_2|\psi_-\rangle$. The Berry phase $\gamma^\pm(k_x)$ for each k_y-subsystem is

$$\gamma^\pm(k_x) = \oint dk_y \mathcal{A}_2^\pm(k_x, k_y). \tag{18}$$

The Berry connection is defined for the conducting and valence bands, respectively, as

$$\mathcal{A}_2^\pm(k_x, k_y) = \langle\psi_\pm(k_x, k_y)|\mathrm{i}\,\partial_{k_y}|\psi_\pm(k_x, k_y)\rangle. \tag{19}$$

4. Eigenmode of the decoupled circular and peanut waveguides

The fundamental mode HE_{11} of a z-invariant straight waveguide can be solved analytically when the cross section is circular [S2], but numerical calculations must be applied for those with peanut-like cross section. The propagation constant of the fundamental mode HE_{11} versus the radius of the circular waveguide is shown in **Figure S1**a. Considering the isotropic geometrical structure and the mode coupling to the p orbital, two degenerate modes, s_1 and s_2, are supported as shown in **Figure S2**.

Based on the coupled mode theory, the antisymmetric and symmetric modes can be built when two monopole resonances are interacted. Here, the antisymmetric mode can be treated as a p orbital. The propagation constant of the p orbital versus the radius of the

constituent circular waveguides is shown in Figure S1b. Due to the degeneracy of the s orbital, the resultant p orbitals also have two different field profiles (p_1 and p_2) as shown in Figure S2. These two p orbitals have nearly identical propagation constants, which can be considered as degeneracy.

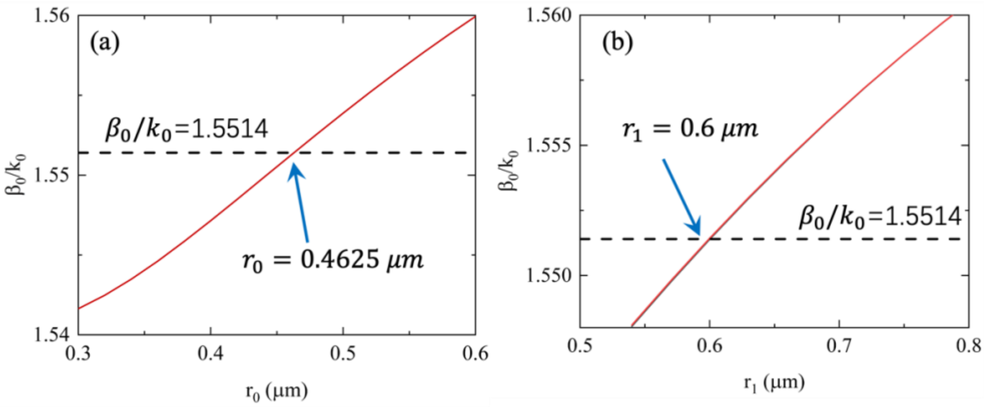

Figure S1. The evolution of the propagation constant β_0 versus the radius of (a) circular waveguide r_0 and (b) the radius of the composed circular waveguide of peanut waveguide r_1. Here, the center-to-center distance is fixed to be d_1 =1 μm (<$2r_1$).

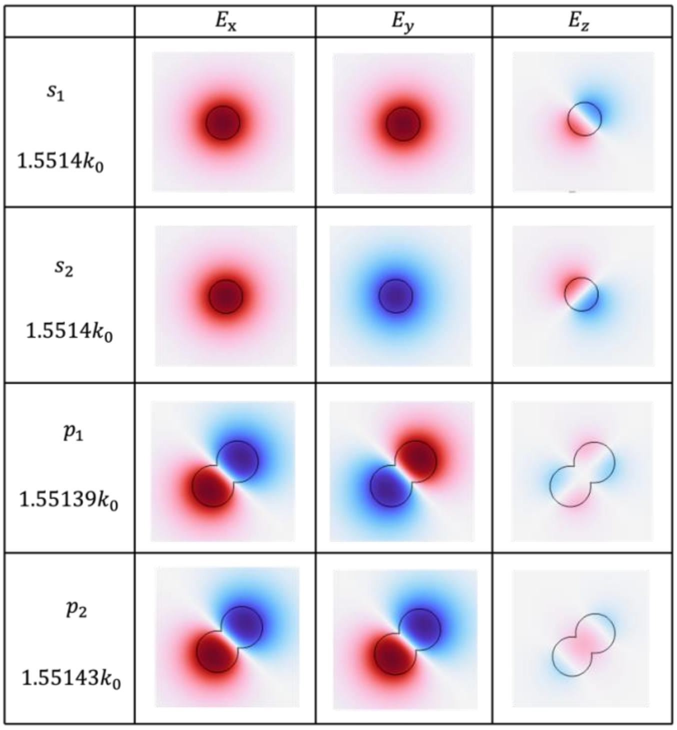

Figure S2. The eigenmode field patterns of the decoupled circular and peanut waveguides.

5. The topological phase diagram with respect to the geometrical dimerization

Table. 1. The topological phases (Mobius semimetal and Weyl semimetals) versus the geometrical parameters. The original lattice configuration is indicated by the blue dashed box. The transformed configuration under specific dimerization is indicated by the red solid box. The yellow box marks the unit cell.

	Möbius	Weyl	
$l_{11} = l_{21} = l_{12} = l_{22}$	$w_{11} = w_{12} > w_{21} = w_{22}$ 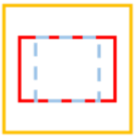	$w_{11} > w_{12}$ $w_{21} < w_{22}$ 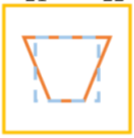	$w_{11} > w_{12}$ $w_{21} < w_{22}$ 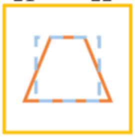
$w_{11} = w_{12} = w_{21} = w_{22}$	$l_{11} = l_{21} > l_{12} = l_{22}$ 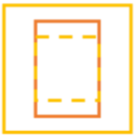	$l_{11} > l_{21}$ $l_{12} < l_{22}$ 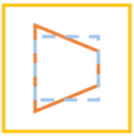	$l_{11} < l_{21}$ $l_{12} > l_{22}$ 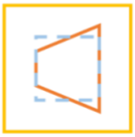
$w_{11} > w_{12}$ $w_{21} < w_{22}$		$l_{11} > l_{21}$ $l_{12} < l_{22}$ 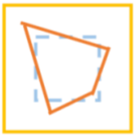	$l_{11} < l_{21}$ $l_{12} > l_{22}$ 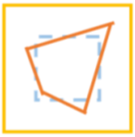
$w_{11} < w_{12}$ $w_{21} > w_{22}$		$l_{11} > l_{21}$ $l_{12} < l_{22}$ 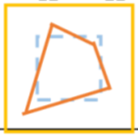	$l_{11} < l_{21}$ $l_{12} > l_{22}$ 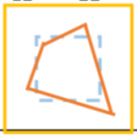

6. Coupled mode theory results for the waveguide array

The coupling amplitudes between the decoupled modes of the circular and peanut waveguides are obtained by the integral of the individual modes [S3], i.e.,

$$t_{mn} = \frac{\omega\epsilon_0}{2}\iint (n_{co}^2 - c_{cl}^2)\mathbf{E}_m^* \cdot \mathbf{E}_n dxdy \qquad (20)$$

where $\mathbf{E}_m$ is the m-th normalized eigenstate of the isolated waveguides. With that, the nearest-coupling strength of the original waveguide lattice without dimerization is $t =$

$0.00027k_0$. Here, the next-nearest coupling strength of the diagonal waveguides (p orbitals) is extremely small, whereas the next-nearest coupling strength of the off-diagonal waveguides (s orbitals) is $0.00002k_0$. Both of them can be neglected. Then, the Hamiltonian of the unperturbed waveguide lattice without dimerization is

$$H_{1,2} = \begin{bmatrix} 1.55139k_0 & t(1-e^{-ik_x}) & t(1+e^{ik_y}) & 0 \\ t(1-e^{ik_x}) & 1.55138k_0 & 0 & -t(1-e^{ik_y}) \\ t(1+e^{-ik_y}) & 0 & 1.55138k_0 & t(1-e^{-ik_x}) \\ 0 & -t(1-e^{-ik_y}) & t(1-e^{ik_x}) & 1.55143k_0 \end{bmatrix} \quad (21)$$

When the dimerization along the y direction [see Figure 4b of the main text)] is introduced, the Hamiltonian is changed to

$$H_{1,2} = \begin{bmatrix} 1.55139 & 0.00027(1-e^{-ik_x}) & 0.00015+0.0005e^{ik_y} & 0 \\ 0.00027(1-e^{ik_x}) & 1.55138 & 0 & -0.00015+0.0005e^{ik_y} \\ 0.00015+0.0005e^{-ik_y} & 0 & 1.55138 & 0.00027(1-e^{-ik_x}) \\ 0 & -0.00027(1-e^{-ik_y}) & -0.00015+0.0005e^{-ik_x} & 1.55143 \end{bmatrix} k_0$$

(22)

In this case, the band structure of the supercell with open boundary condition along the y direction and periodic boundary condition along the x direction is shown in **Figure S3(**a). This is consistent with the numerical results obtained by FEM.

For the case shown in Figure 4c of the main text, the corresponding Hamiltonian obtained by the CMT is

$$H_{1,2} = \begin{bmatrix} 1.55139 & 0.00027(1-e^{-ik_x}) & 0.00015+0.0005e^{ik_y} & 0 \\ 0.00027(1-e^{ik_x}) & 1.55138 & 0 & 0.0005-0.00015e^{ik_y} \\ 0.00015+0.0005e^{-ik_y} & 0 & 1.55138 & 0.00027(1-e^{-ik_x}) \\ 0 & -0.00027(1-e^{-ik_y}) & 0.0005-0.00015e^{-ik_x} & 1.55143 \end{bmatrix} k_0$$

(23)

Then the bulk and edge bands from Eq. (23) are shown by the blue dashed curves in Figure S3(b). There is a slight offset between the results from the CMT and the FEM (solid curves in Fig3(b). This is due to the existence of the next-nearest-neighboring coupling and a slight mismatch between the propagation constants of the peanut and circular waveguides. Notice that the CMT model does not account for the next-nearest-neighboring coupling.

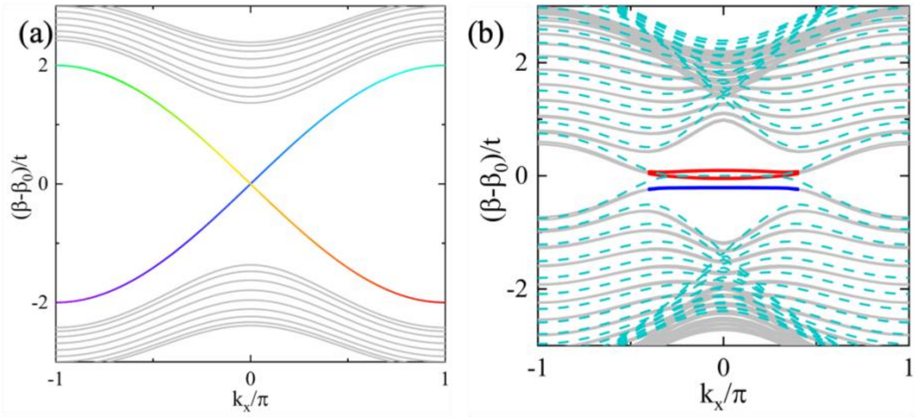

Figure S3. CMT-based dispersions for the waveguide supercell structures with open boundary condition along the y direction and periodic boundary condition along the x direction. (a) Möbius insulator for the case with $w_{11} = w_{12} = w_{21} = w_{22} = a/2$, $l_{11} = l_{21} = a/2 + 0.03a$ and $l_{12} = l_{22} = a/2 - 0.03a$. (b) Weyl-like semimetal phases for dimerization along the y direction: $l_{11} = l_{22} = a/2 + 0.03a$ and $l_{12} = l_{21} = a/2 - 0.03a$. The blue dashed curves are results from the CMT model, i.e., Eq. (23).

7. The effect of the next-nearest-neighbor coupling for the Weyl Hamiltonian

Within the main text, Equation (8) describes the Weyl Hamiltonian $\mathcal{H}_{\mathrm{IV}}(\mathbf{k})$ corresponding to the gauge configuration shown in Figure 1f and Figures 2e,f, with the next-nearest-neighbor (NNN) coupling neglected. Notice that, in our waveguide arrays, each waveguide hosts two orthogonal eigenstates, as schematically shown in Figure S4(a). In this case, the tight-binding Hamiltonian can be block diagonalized as $\mathcal{H}_{\mathrm{IV}}^{'}(\mathbf{k}) = \mathrm{Diag}[\mathcal{H}_{\mathrm{IV}}^{\mathrm{A}}(\mathbf{k}), \mathcal{H}_{\mathrm{IV}}^{\mathrm{B}}(\mathbf{k})]$, where $\mathcal{H}_{\mathrm{IV}}^{\mathrm{A}}(\mathbf{k})$ and $\mathcal{H}_{\mathrm{IV}}^{\mathrm{B}}(\mathbf{k})$ are, respectively, for the mode couplings among the eigenmodes with symemtries featured by the red and blue arrows. When being taken into account, however, the NNN coupling t_3 between A and D, B and C for $\mathcal{H}_{\mathrm{IV}}^{\mathrm{A(B)}}(\mathbf{k})$ representing a perturbation $\mathcal{H}_{\mathrm{NNN}}^{\mathrm{A(B)}}(\mathbf{k})$ $(4 \times 4$ matrix) shall be incorporated,

$$\mathcal{H}_{\mathrm{NNN}}^{\mathrm{A(B)}}(\mathbf{k}){=}{-}\mathrm{t}_3\big(1 + \cos(\mathrm{k_x} + \mathrm{k_y})\big)\Gamma_2\Gamma_4 - \mathrm{t}_3 \sin(\mathrm{k_x} + \mathrm{k_y})\,\Gamma_3\Gamma_4 \qquad (24)$$

Besides, the NNN coupling t_4 between the eigenmodes featured by different arrows in A and D, B and C shall also be considered. These perturbations are denoted by $\mathcal{H}_{\mathrm{NNN}}^{\mathrm{AB}}(\mathbf{k})$ and $\mathcal{H}_{\mathrm{NNN}}^{\mathrm{BA}}(\mathbf{k})$

$$\mathcal{H}_{\mathrm{NNN}}^{\mathrm{AB}}(\mathbf{k})=-\frac{t_4}{2}\left(1+e^{-i(k_x+k_y)}\right)\Gamma_2\Gamma_4+\frac{t_4}{2}e^{i(k_x+k_y)}\Gamma_3\Gamma_4 \tag{25}$$

Here, $\mathcal{H}_{\mathrm{NNN}}^{\mathrm{BA}}=(\mathcal{H}_{\mathrm{NNN}}^{\mathrm{AB}})^{\dagger}$. The tight-binding Hamiltonian now becomes

$$\mathcal{H}_{\mathrm{IV}}^{'}(\mathbf{k})=\begin{bmatrix}\mathcal{H}_{\mathrm{IV}}^{\mathrm{A}}(\mathbf{k})+\mathcal{H}_{\mathrm{NNN}}^{\mathrm{A}}(\mathbf{k}) & \mathcal{H}_{\mathrm{NNN}}^{\mathrm{AB}}(\mathbf{k})\\ \mathcal{H}_{\mathrm{NNN}}^{\mathrm{BA}}(\mathbf{k}) & \mathcal{H}_{\mathrm{IV}}^{\mathrm{B}}(\mathbf{k})+\mathcal{H}_{\mathrm{NNN}}^{\mathrm{B}}(\mathbf{k})\end{bmatrix} \tag{26}$$

Figure S4(b) shows the band structure for turning off (red solid line, $t_3=0$) and on (black dashed lines, $t_3=0.1$) of $\mathcal{H}_{\mathrm{IV}}^{\mathrm{A(B)}}(\mathbf{k})$, assuming that $\mathcal{H}_{\mathrm{NNN}}^{\mathrm{AB}}(\mathbf{k})$ is completely vanishing. Clearly, the Weyl point remains in presence. However, Figure S4(c) shows the band gap opens when the $\mathcal{H}_{\mathrm{NNN}}^{\mathrm{AB}}(\mathbf{k})$ and $\mathcal{H}_{\mathrm{NNN}}^{\mathrm{BA}}(\mathbf{k})$ terms are turned on (black dashed lines, $t_4=0.1$). Compared to the case with $t_4=0$ [red solid lin in Fig. S4(b)], it is clearly seen that a minor gap opens at the Weyl point in the ΓX direction. Notice that for the case in presence of the NNN coupling, the doubly degenerated bands confirm the numerical findings displayed in Figure 5(b) within the main text.

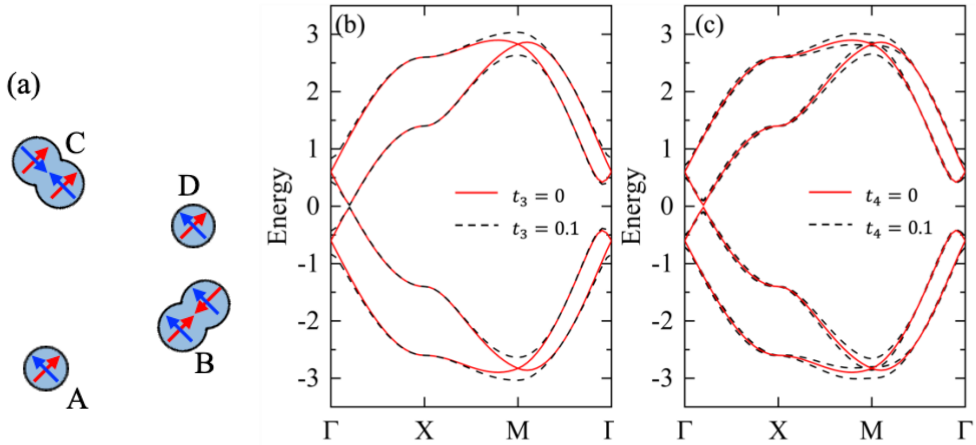

Figure S4. Schematic of the waveguides array and the band structure calculated using the tight-binding model considering the NNN coupling between the off-diagonal sites. (a) Each waveguide hosts two orthogonal modes, represented by the arrows featuring the E_z fields. (b) The red solid line is for the Hamiltonian $\mathcal{H}_{\mathrm{IV}}^{\mathrm{A(B)}}(\mathbf{k})$ without NNN coupling, i.e., $t_3=0$, the black dashed line is for the Hamiltonian $\mathcal{H}_{\mathrm{IV}}^{\mathrm{A(B)}}(\mathbf{k})+\mathcal{H}_{\mathrm{NNN}}^{\mathrm{A(B)}}(\mathbf{k})$ with $t_3=0.1$. (c) The red solid line is for the Hamiltonian $\mathcal{H}_{\mathrm{IV}}^{'}(\mathbf{k})$ without NNN coupling, i.e., $t_4=0$, the black dashed line is for the Hamiltonian $\mathcal{H}_{\mathrm{IV}}^{'}(\mathbf{k})$ with $t_4=0.1$.

8. The field profile for the various edge states

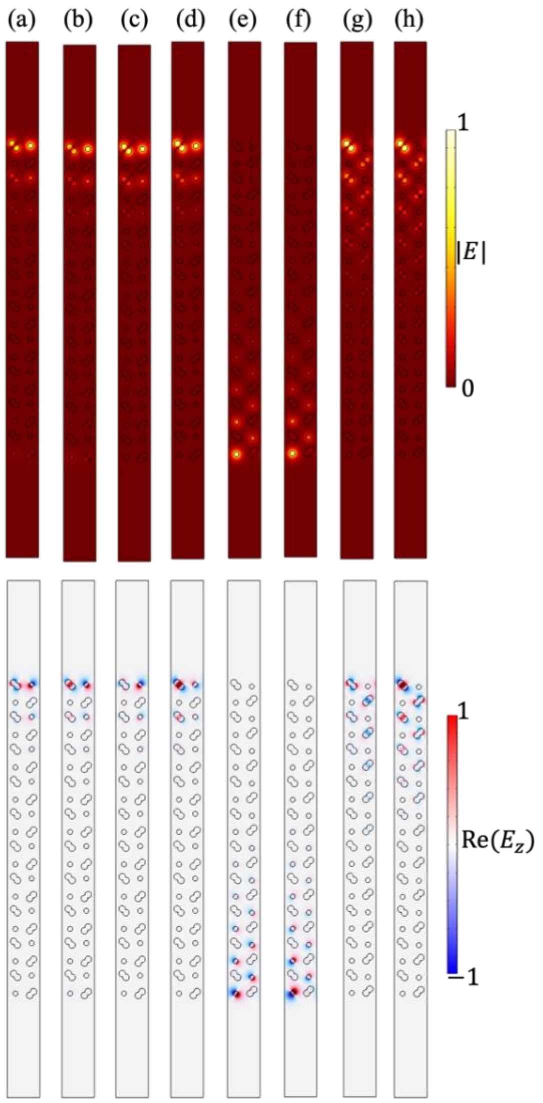

Figure S5. The field pattern $|\mathrm{E}|$ and $\mathrm{Re}(\mathrm{E_z})$ for the edge states corresponding to (d-k) of Figure 5 in the original text, respectively. The top panel is for $|E|$, the bottom panel is for $\mathrm{Re}(E_z)$.

9. The eigenvalues of the projective translation symmetry

The edge state can be written in the form as $|\psi(\boldsymbol{k})\rangle = e^{-k_y y}(\phi_A, \phi_B, \phi_C, \phi_D)^T$, where ϕ_i represents the i-th component of the sublattice. Primitive translation operator $\mathrm{L_x}$ acting on the eigenstates $\psi(\boldsymbol{k})$ yields

$$\hat{\mathrm{L}}_{\mathrm{x}}|\psi(\boldsymbol{k})\rangle = e^{-k_y y}(\phi_B, \phi_A e^{ik_x}, -\phi_D, -\phi_C e^{ik_x})^T = \pm i e^{ik_x/2}|\psi(\boldsymbol{k})\rangle \quad (26)$$

where $\pm i e^{ik_x/2}$ are the eigenvalues ℓ_s of $\mathrm{L_x}$. Therefore, the eigenvalues ℓ_s can be obtained in terms of $\ell_s = -\phi_D/\phi_C$ or $\ell_s = \phi_B/\phi_A$.

REFERENCES

S1. Zhao Y. X., Huang Y.-X., and Yang S. A., Z_2 -Projective Translational Symmetry Protected Topological Phases, *Phys. Rev. B* **102**, 161117 (2020).

S2. Pollock C. R. and Lipson M., Integrated photonics. (Kluwer Academic, 2010).

S3. Liu Z. Z., Zhang Q., Chen Y. T., and Xiao J. J., General coupled mode analysis of geometry symmetric waveguide array with non-uniform gain and loss, *Photonics Research* 5, 57-63 (2017)
